# Restoring immune tolerance in pre-RA: immunometabolic dialogue between gut microbiota and regulatory T cells

**DOI:** 10.3389/fimmu.2025.1565133

**Published:** 2025-03-20

**Authors:** Anqi Gao, Ruihe Wu, Yanfei Mu, Ruqing Jin, Saixin Jiang, Chong Gao, Xiaofeng Li, Caihong Wang

**Affiliations:** ^1^ Department of Rheumatology, The Second Hospital of Shanxi Medical University, Taiyuan, Shanxi, China; ^2^ Department of Rheumatology, Shanxi Key Laboratory of Rheumatism Immune Microecology, Taiyuan, Shanxi, China; ^3^ Department of Rheumatology, Shanxi Precision Medical Engineering Research Center for Rheumatology, Taiyuan, Shanxi, China; ^4^ Pathology, Joint Program in Transfusion Medicine, Brigham and Women’s Hospital/Children’s Hospital, Harvard Medical School, Boston, MA, United States

**Keywords:** rheumatoid arthritis, pre-RA, regulatory T cell, gut microbiota, immunometabolism

## Abstract

Rheumatoid arthritis (RA) is a complex chronic autoimmune disease that remains incurable for most patients. With advances in our understanding of the disease’s natural history, the concept of pre-RA has emerged as a window of opportunity to intervene before irreversible joint damage occurs. Numerous studies have indicated that the key step driving autoimmunity in early pre-RA lies at an extra-articular site, which is closely related to the regulatory T (Treg) cell-established immune tolerance to the gut microbiota. The intricate immunometabolic crosstalk between Treg cells and the gut microbiota is beginning to be understood, with the re-recognition of Treg cells as metabolic sensors in recent years. In the future, deciphering their immunometabolic dialogue may help to elucidate the underlying mechanisms of pre-RA. Identifying novel biological pathways in the pre-RA stage will bring insights into restoring immune tolerance, thereby potentially curing or preventing the onset of RA.

## 1 Introduction

Rheumatoid arthritis (RA) is a highly disabling chronic autoimmune disorder (1). Approximately 17.6 million people worldwide are living with RA, as of 2021, which not only affects patients’ quality of life but also imposes a serious burden on families and society (2). Due to the unclear pathogenesis, current therapies struggle to address the underlying immune mechanisms of RA, and thus it remains a lifelong incurable disease for most patients. Encouragingly, studies have found that early diagnosis and management can prevent or slow the progression of joint damage in 90% of RA patients, suggesting a perceived “window of opportunity” (3). The focus of early intervention research has now shifted from diagnosed RA to a stage at risk of developing RA, termed “pre-RA”, characterized by aberrant autoantibodies and other biomarkers before the onset of clinically apparent inflammatory arthritis (IA) (4, 5). A better understanding of pre-RA has been proposed as a current unmet need in the rheumatology field (6). Identifying individuals in the pre-RA phase for intervention may potentially delay or even prevent the onset of clinically-apparent IA (7).

The key step in the transition from pre-RA to clinically-apparent IA is considered to be the disruption of immune tolerance. Although the critical biological pathways that drive the initial destruction of tolerance remain unknown, evidence such as imaging or synovial biopsy in at-risk individuals strongly indicates that autoimmunity arises outside the joints and may be associated with mucosal events (8–10). This mucosal origin hypothesis has driven a series of findings suggesting that the oral mucosa, respiratory mucosa, and gut mucosa may serve as potential sites of RA pathogenesis (11–13). Notably, the prominent gut microbiota dysbiosis observed in RA patients and its association with disease severity has led to growing interest in the gut mucosa as a site of disease initiation (14, 15). Sustaining the reciprocal relationship between the gut microbiota and the host relies primarily on regulatory T (Treg) cells, as they establish tolerance to the gut microbiota during the early stages of life development (16, 17). Taken together, the earliest step leading to immune tolerance breakdown in pre-RA may be the disruption of Treg cell-induced tolerance to gut microbiota due to dysbiosis. However, the exact mechanisms remain unclear.

Immunometabolism refers to the modulation of immune cell responses through metabolic pathways in both health and illness conditions, which is becoming an increasingly valuable area for understanding the pathogenesis of autoimmune diseases (18). With advancements in immunometabolism studies, Treg cells have been re-recognized as key metabolic sensors capable of undergoing metabolic reprogramming in response to influences of immune microenvironments (19). The unique sensitivity to metabolic signals allows Treg cells to rapidly sense energy in key metabolic sites, especially the gut, and enrich in them. Changes in massive microbial metabolic signals can be sensed by Treg cells and translated into immune regulation (19). In turn, immune signals produced by gut Treg cells play a crucial role in shaping and protecting the ecology of the gut microbiota (20). Therefore, a regulatory circuit is formed between Treg cells and gut microbiota through immunometabolic dialogue to maintain host-microbe symbiosis and immune homeostasis. Disorders of gut microbiota may affect Treg cell-induced immune tolerance by altering local microenvironmental metabolism, thus triggering autoimmunity initiation. Further deciphering the immunometabolic dialogue between gut microbiota and Treg cells may provide new therapeutic opportunities based on Treg cells, gut microbiota, and metabolites for RA.

Prevention of RA has always been a major clinical concern, while intervention of individuals in the pre-RA stage is a relatively novel concept. In this review, we summarize the current understanding of pre-RA. By discussing the present knowledge of immune tolerance mechanisms, we propose that autoimmunity activation in pre-RA may start with the disruption of Treg cell-induced tolerance to gut microbiota. Integrating new insights from the immunometabolism field, we suggest that the mechanisms of immune tolerance destruction in pre-RA may be related to metabolic pathways. We further describe the regulatory circuit formed between Treg cells and gut microbiota through immunometabolic dialogue. Collaboration among research teams with different capabilities and expertise will help unravel their complex relationship. Hopefully, this will enable the early identification of at-risk individuals and the finding of new targets for reversing pathological states to a healthy condition.

## 2 Loss of immune tolerance in pre-RA

### 2.1 Pre-RA may be a better therapeutic window of opportunity

**Figure 1:**
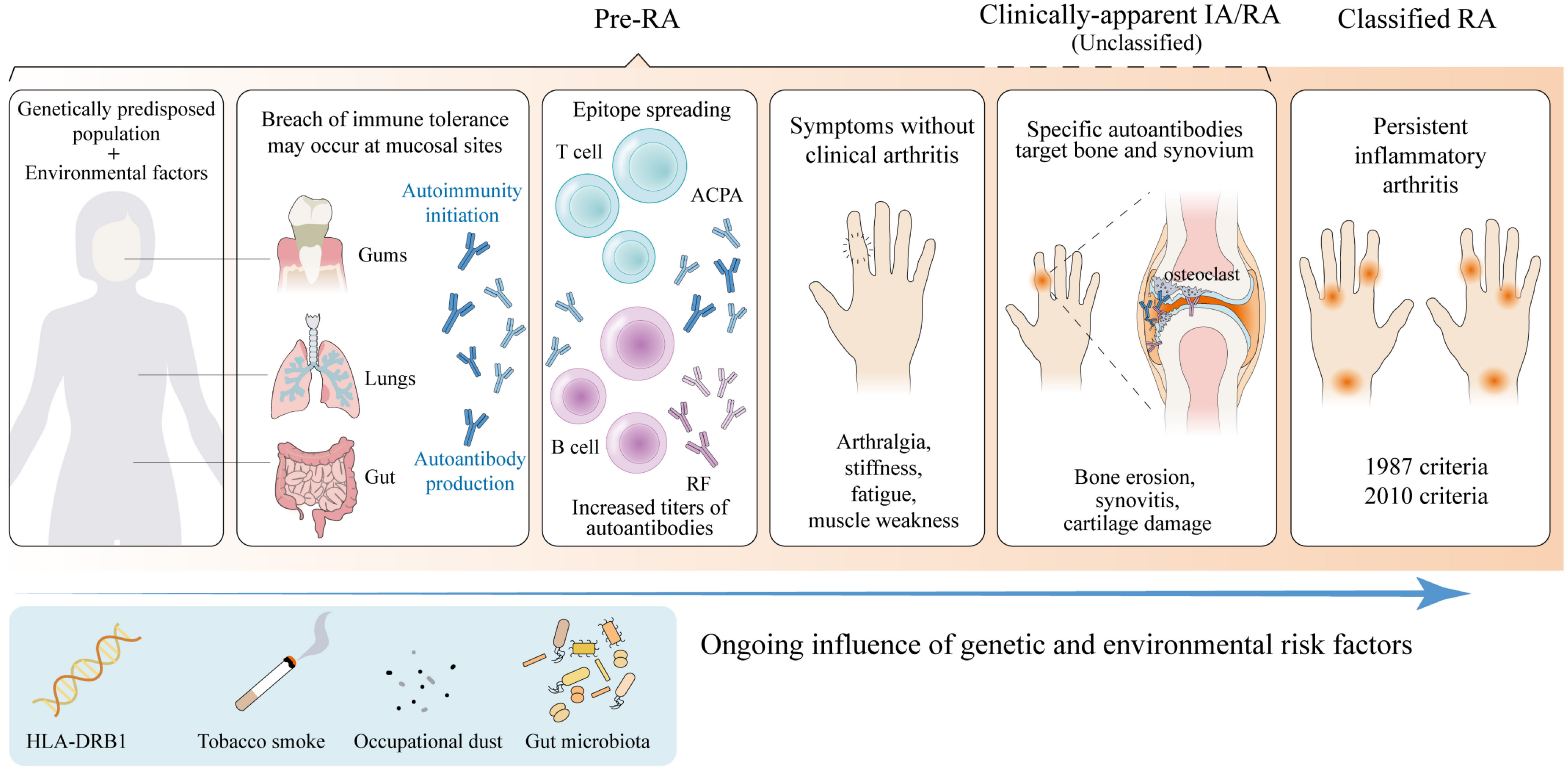
Phases of rheumatoid arthritis progression. The progression of rheumatoid arthritis (RA) is considered to be a multi-step process. Under the interaction of various environmental and behavioral risk factors, genetically predisposed populations first experience disruption of immune tolerance at mucosal sites (21). Originally, an early response to a limited number of autoantigens occurs, followed by epitope spreading and increased autoantibody titers (24). With innate and adaptive immune responses amplifying, specific autoantibodies against modified antigens target synovial and bone compartments, triggering local inflammatory responses and eventually culminating in clinically-apparent inflammatory arthritis (IA)/RA (25). There is some controversy about whether unclassified arthritis can be categorized as part of Pre-RA. All of the above stages do not necessarily occur in all patients who eventually develop RA, nor do they necessarily occur in the same order. *RF, rheumatoid factor; ACPA, anti-citrullinated protein antibody. (Created with Adobe Illustrator)*.

RA is a complicated chronic autoimmune disease with progression considered to be a multi-step process. Under the interaction of various environmental and behavioral risk factors, genetically predisposed populations first experience a breakdown of immune tolerance, possibly at mucosal sites, triggering autoimmunity initiation years before the onset of apparent clinical manifestations (21, 22). Originally, an early response to a limited number of autoantigens occurs, followed by epitope spreading and increased autoantibody titers (23, 24). With innate and adaptive immune responses amplifying, specific autoantibodies against modified antigens target synovial and bone compartments, triggering local inflammatory responses and eventually culminating in IA/RA (25). All of the above stages do not necessarily occur in all patients who eventually develop RA, nor do they necessarily occur in the same order (**Figure 1**). Studies show that about half of RA patients have positive rheumatoid factor (RF) and/or anti-citrullinated protein antibody (ACPA) measurements for a median of 4.5 years before the symptoms appeared (26). Moreover, a pre-clinical proteomic signature has been identified through analyzing serum samples from first-degree relatives of North American RA patients, further demonstrating that serum proteomic differences between those who progressed to RA and other high-risk individuals exist years before the disease onset, regardless of ACPA status (5). This period of RA development, characterized by aberrant autoantibodies and other biomarkers before the emergence of clinically-identifiable IA, can be termed “pre-RA” (4, 27).

The visions of early diagnosis and intervention have shifted the focus of research in recent years from patients with established RA to those at risk of developing RA. With a growing understanding of RA progression, the pre-RA status may represent a better therapeutic window of opportunity, which indicates a chance of managing the disease before substantial tissue damage develops (7, 28). From this perspective, several clinical prevention trials have been conducted to provide immunological interventions in pre-RA individuals (29–31). The TREAT EARLIER study showed that methotrexate did not prevent the progression of clinical arthritis in at-risk individuals but modified the disease course compared to placebo (31). The recent ARIAA and APIPPRA trials have demonstrated that treatment with abatacept reduces inflammation, clinical symptoms, and the onset of RA in individuals at high risk, offering hope for RA prevention (29, 30). Current prevention trials are evaluating drugs that have been used in clinically-apparent RA, however, the biological pathways underlying pre-RA progression may differ from those present in established RA. Further studies addressing immune mechanisms in the pre-RA phase will help identify novel biomarkers and therapeutic targets.

Now the comprehension of immune dysregulation in pre-RA is rapidly advancing. The key step in the progression of pre-RA to clinically-apparent RA is considered to be the disruption of immune tolerance and thereby the initiation of autoimmunity.

### 2.2 Immune tolerance disruption as a pivotal step of pre-RA

Loss of tolerance to autoantigens or harmless antigens during pre-RA is an early and central stage in RA progression. Early epidemiological studies back to the 90’s identified the presence of RFs precedes synovitis, suggesting that self-tolerance breakdown antedates clinically-apparent RA (32). Immune tolerance refers to a state of immune system unresponsiveness to certain molecules or tissues, comprising central and peripheral tolerance. The former is the primary way the immune system distinguishes between self and non-self, while the latter prevents the immune system from overreacting to various environmental factors (33). Although the specific mechanisms leading to immune tolerance breakdown are currently obscure, pre-RA is thought to be driven by a combination of genetic predisposition and multiple acquired environmental triggers (34). Potential environmental factors include smoking, various infections, and especially microbiota alterations, contributing to immune tolerance failure to certain autoantigens, with the activation of specific T and B cells and subsequent overproduction of abnormal autoantibodies, propelling the development of pre-RA to clinically-identifiable RA (22, 35, 36).

Current studies have yielded some insights into the mechanisms of immune tolerance destruction in pre-RA. In the sputum of at-risk first-degree relatives of RA patients, elevated levels of neutrophil extracellular trap (NET) complexes were observed, correlating with the production of mucosal anti-cyclic citrullinated peptide (anti-CCP) antibodies (37). These findings suggest that dysregulated NET formation in the inflamed airways of individuals at-risk of RA may drive autoantibodies generation in the preclinical phase. Impaired early B cell tolerance has long been recognized as an influential factor in the pathogenesis of RA. A study of RA patients revealed that autoreactive B cells fail to be cleared in all six patients enrolled, accounting for 35% to 52% of the mature naïve B cell area (38). Moreover, an increased proportion of multi-reactive antibodies expressed by RA B cells in certain patients was found, indicating an early deficiency in central B cell tolerance (38). Nevertheless, these mechanisms are all associated with the production of autoantibodies, thus challenging to explain the pathogenesis of seronegative RA. T cell tolerance is now gaining increasing attention in the study of RA pathogenesis, particularly for Treg cells, an important component of immune tolerance maintenance.

Treg cells represent one subgroup among T cells expressing CD4, CD25 and Forkhead Box P3 (FoxP3). Depending on the generation site, Treg cells are divided into two subpopulations, thymic Tregs (tTregs) and peripheral Tregs (pTregs), both associated with the establishment of peripheral tolerance (39). Multiple studies have revealed that both depletion and dysfunction of Treg cells play crucial roles in the pathogenesis of RA. Compared to healthy individuals, the frequency of Treg cells in the peripheral blood was markedly lower in RA patients (40). A study found that although the number of Treg cells in synovial fluid of RA patients was increased compared to that of osteoarthritis patients, they failed to suppress the activation and maturation of dendritic cells (DCs), suggesting an impaired inhibiting function (41). RA is closely associated with a disturbed T helper-17 (Th17)/Treg balance (42, 43). The impairment of Treg cell number and function skews the Th17/Treg balance towards pro-inflammatory Th17 cells, which may be the main mechanism for Treg cell involvement in the development of RA (43). Studies have revealed significantly low absolute counts of Treg cells and high Th17/Treg cell ratio in patients with seropositive undifferentiated arthritis (SUA), indicating the potential role of Treg cells in the pre-RA stage (44). Besides, a decrease in Treg cell percentage was also found in patients with seronegative RA and was lower than that in patients with seropositive RA, suggesting that Treg cell abnormalities may also play a role in seronegative RA development (45). In conclusion, the impairment of Treg cells may be a pivotal event in the disruption of immune tolerance in pre-RA, yet the upstream mechanisms have rarely been investigated. Previous studies have revealed that many patients with elevated RA-associated autoantibodies show no evidence of synovitis upon physical examination, imaging, or synovial biopsy, strongly suggesting that autoimmunity arises outside the joints (8, 9, 46). There is growing evidence that the activation and propagation of autoimmunity in pre-RA may be associated with mucosal events, particularly alterations in the mucosal microbiota (10, 47). The maintenance of a reciprocal relationship between the gut microbiota and the host immune system ensures the homeostasis of most microbial communities and non-aggressive immune cell compartments, in which Treg cells play a critical role (17, 48). Gut dysbiosis may impair Treg cell-mediated immune tolerance, leading to a disruption of mucosal immune homeostasis and triggering initial inflammation.

### 2.3 Breach of tolerance to the gut microbiota may be the earliest failure

There have been numerous studies showing dysbiosis in RA patients. Comparisons of the gut microbiota in RA patients and HC revealed that the gut microbiota diversity was reduced in RA patients, with an abundance of gram-positive bacteria and a decrease in gram-negative bacteria (14, 15). Compared to patients with established RA or other chronic arthritis, stool samples from patients with newly developed RA showed an expansion of *Prevotella copri* and a decline in *Bacteroides* (49). More interestingly, a cross-sectional analysis indicated that the fecal microbiota of individuals at risk for RA had higher levels of *Prevotella* spp compared to first-degree relatives, which proved that dysbiosis occurs at the pre-RA stage (50). In addition, research with collagen-induced arthritis in mice has shown that alterations of gut flora through oral antibiotics exacerbated disease severity compared to controls (51). There is also evidence supporting that gut microbiota is associated with RA via affecting the balance between Treg cells and Th17 cells (52). Therefore, the dysbiosis of certain gut microbiota leads to changes in the host immune profile, which may be the first step of mucosal immune tolerance disruption.

Gut Treg cells induce tolerance to the gut microbiota, meanwhile, their differentiation, migration, and maintenance are regulated by specific signals offered by the local environment, particularly antigens and immunomodulatory molecules continuously provided by certain kinds of microbiota (53). Experimental evidence in recent years is gradually linking immunity and metabolism. New findings suggest that the key route by which gut microbiota disorders affect Treg cell-induced immune tolerance may be metabolic pathways.

## 3 Metabolic changes affect immune tolerance

### 3.1 Immunometabolism and metabolic reprogramming

Immunometabolism refers to the modulation of immune cell responses through metabolic pathways in both health and illness conditions (18). There is growing evidence supporting metabolism as an important factor in regulating immune cell phenotype and function (54). Metabolic reprogramming describes the process of allowing cells to meet their energy demands by altering metabolic pathways that control cellular energetics and biosynthesis, which has long been considered a hallmark of tumor cells (55). As our understanding of immunometabolism advances, metabolic reprogramming is now being brought into the realm of autoimmune diseases. Studies have demonstrated that immune cells exhibit significant metabolic changes upon activation that affect their immune functions (56). Performing metabolic reprogramming is essential for their normal function while may also driving disorders. Furthermore, the role of metabolites along with their capacity to modulate signaling pathways is under increasing investigation, and researchers are attempting to establish a link between environmental elements and the pathogenic actions of immune cells in RA (57).

Recent research has highlighted the involvement of metabolic signaling in the maintenance and function of Treg cells. It is proposed that Treg cells may act as key metabolic sensors, sensing cues in the core of metabolic centers by recording fluctuations of nutrients and peripheral energy sensors (19). On the one hand, Treg cells can translate local metabolic signals into the regulation of immune tolerance (19). On the other hand, nutritional condition variations in local tissues can drive metabolic reprogramming of Treg cells, affecting their stability and function (39). The metabolic features of Treg cells are highly heterogeneous from other T cells. Typically, proinflammatory cells like effector T cells and M1 macrophages employ glycolysis as a rapid method of energy production, whereas Treg cells rely primarily on fatty acid oxidation (FAO) and oxidative phosphorylation (OXPHOS) (58). Moreover, Treg cells exhibit heterogeneous metabolic profiles in different environments, depending on the availability of nutrients and the activation status of the cells. They participate in a nutrient-sensing mechanism to adjust to internal and external environmental cues and perform metabolic reprogramming to retain their activity (39). Under homeostatic conditions, FoxP3 is expressed normally and most Treg cells are phenotypically and functionally stable (59). However, in pathological conditions, alterations in the immune microenvironment impact nutrient availability, compelling Treg cells to undergo metabolic reprogramming, which in turn affects their inhibiting function (39). In the inflammatory environment of RA, Treg cells can sense metabolic cues and inevitably undergo metabolic reprogramming in response to environmental pressures, which may alter their mediated immune tolerance.

### 3.2 Metabolic alterations in RA immune microenvironment affect Treg cell-induced immune tolerance

**Figure 2:**
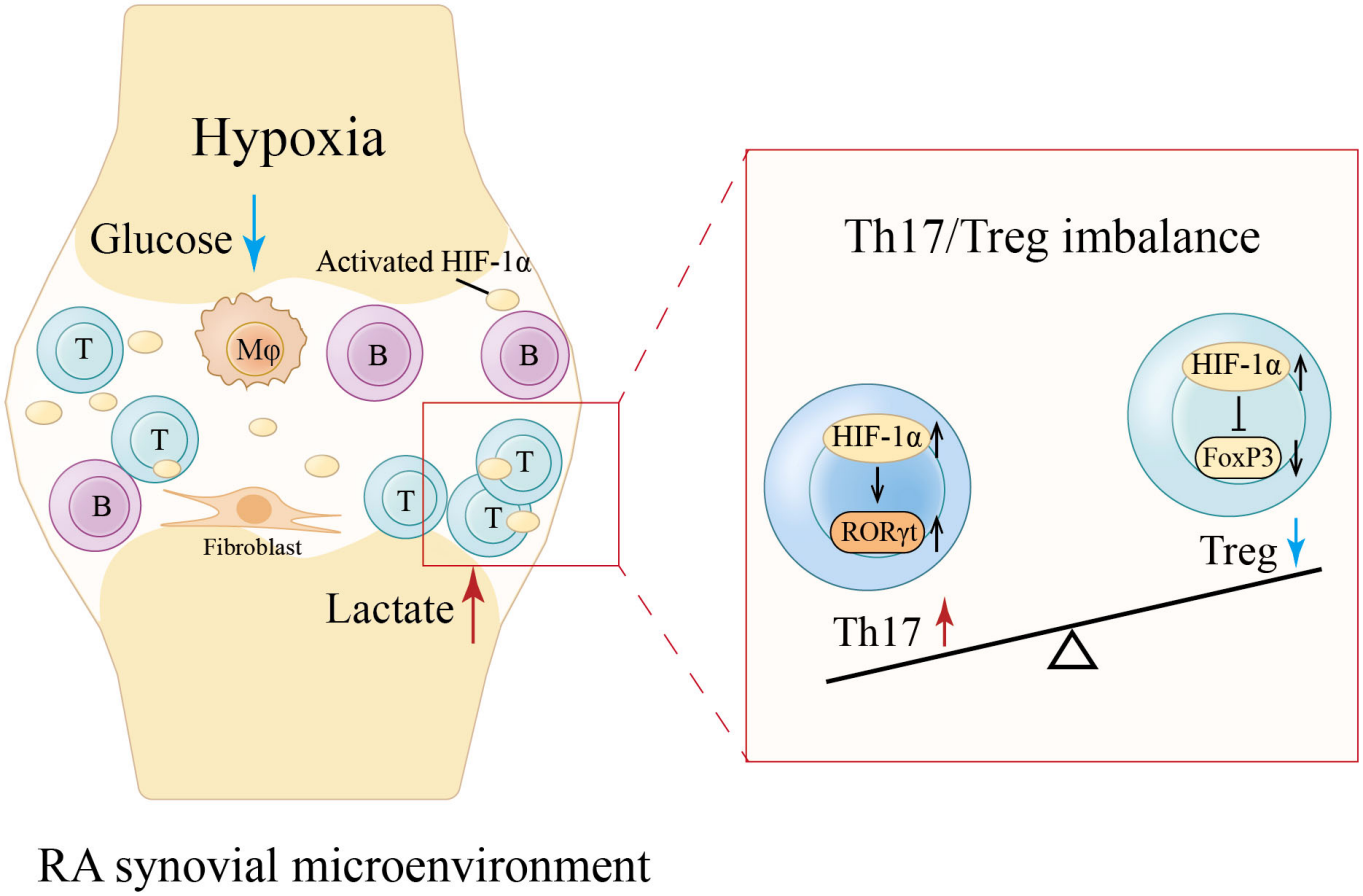
Hypoxia-induced Th17/Treg imbalance in RA synovial microenvironment. The chronic activation of various cells within RA synovium forms a microenvironment lacking oxygen and glucose yet rich in lactate (60). Hypoxia activates and enriches hypoxia-inducible factor (HIF)-1α in RA synovial fluid (61). HIF-1α-dependent induction of RORγt along with FoxP3 degradation is detrimental to Treg cells and promotes Th17 cell generation, leading to a Th17/Treg imbalance (62). *Mφ, Macrophage. (Created with Adobe Illustrator)*.

T cells, B cells, macrophages, and stromal cells within RA inflamed tissues are chronically activated and in a state of high metabolic stress, forming a microenvironment lacking oxygen and glucose yet rich in lactate (60). A consequence of hypoxia is hypoxia-inducible factor (HIF)-1α activation. HIF-1α is enriched in RA synovial fluid and participates in RA pathogenesis through increasing vasoproliferation, oxidative damage, inflammation, apoptosis, and cartilage invasion (61). Driven by a joint hypoxic microenvironment, HIF-1α-dependent induction of RORγt together with FoxP3 degradation is damaging to Treg cells and promotes Th17 cell generation (62) (**Figure 2** **).** This suggests that altered metabolism in the RA synovial microenvironment affects Th17/Treg axis, providing a feed-forward mechanism to amplify inflammation. However, it is unknown how the pre-RA immune microenvironment might shape the metabolic features of Treg cells.

As key metabolic sensors, Treg cells exhibit exceptional sensitivity to systemic metabolic signals, rapidly sensing energy in key metabolic tissues and enriching in them (19). The gut is considered a central region of metabolism and a large population of phenotypically and functionally distinct Treg cells is indeed observed in its lamina propria (LP) (63–65). Most nutritional and non-nutritional compounds in the human gut need to be fermented and catabolized by gut microbiota to produce a series of essential metabolites (66). In the gut microenvironment, Treg cells are exposed to those metabolic signals and perceive their changes, converting them into immune regulation (19). Thus, gut microbiota disorders in the pre-RA stage may affect Treg cell-induced immune tolerance by altering microenvironmental metabolism, which may serve as a major mechanism of pre-RA.

## 4 Gut microbiota regulates Treg cells through metabolic pathways

With the gut being home to up to 80% of the body’s immune cells and the microbiota containing nearly 150 times more genes compared to the host genome, it is not a surprise that gut microbiota metabolites play a significant role in influencing immune cells and regulating immune signals (66). New research highlights the complex interactions between gut microbiota and Treg cells through metabolic pathways that have major implications for immune responses and diseases.

### 4.1 Gut microbiota metabolites regulate Treg cells

Numerous gut microbiota metabolites are involved in regulating Treg cells, such as short-chain fatty acids (SCFAs), bile acids (BAs), and tryptophan (Trp) metabolites (67).

**Figure 3:**
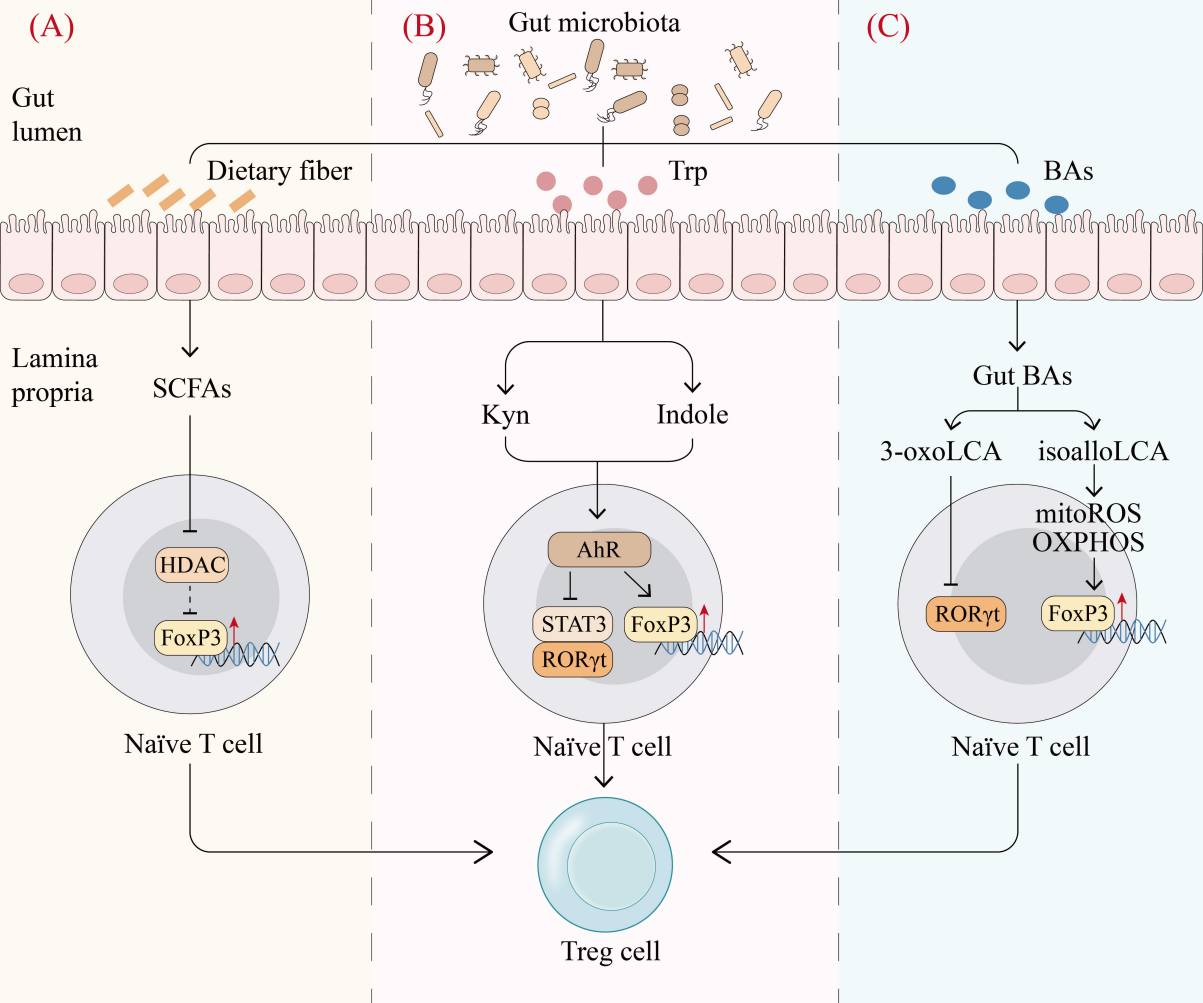
Gut microbiota metabolites regulate Treg cell production. **(A)**, Fermentation of dietary fiber by gut microbiota produces short-chain fatty acids (SCFAs), which can promote the differentiation of naïve T cells into Treg cells by inhibiting histone deacetylase (HDAC) and thereby inducing Forkhead Box P3 (FoxP3) (70). **(B)**, Gut microbiota catabolizes tryptophan (Trp) to produce mainly indole and kynurenine (Kyn) to activate the aryl hydrocarbon receptor (AhR). The activation of AhR upregulates FoxP3 and downregulates ROR γ t and STAT3 to promote the differentiation of naïve T cells into Treg cells (84). **(C)**, Primary bile acids (BAs) are converted to various gut BAs by gut commensal bacteria. Among gut BAs, isoalloLCA promotes Treg cell differentiation by enhancing OXPHOS and mitochondrial reactive oxygen species, which upregulates FoxP3 through histone acetylation. In contrast, 3-oxoLCA directly inhibits the transcription factor RORγt, which favors the differentiation of naïve T cells into Treg cells (74). *mitoROS*, mitochondrial reactive oxygen species*. (Created with Adobe Illustrator)*.

SCFAs are produced mainly by bacterial fermentation of indigestible carbohydrates like dietary fiber and possess immune regulatory properties (68). The balance of fatty acids (FAs) has a significant impact on CD4^+^ T cells, especially on Th17 and Treg cell production. SCFAs can induce FoxP3 through histone deacetylase (HDAC)-dependent manner to promote extrathymic Treg cell development, while long-chain fatty acids (LCFAs) promote the differentiation of Th1 and Th17 cells through MAPKs p38 and JNK1 (69–71) (**Figure 3A**). Accordingly, it was observed that mice fed SCFAs exhibited enhanced differentiation and proliferation of Treg cells, which protected them from inflammation, whereas mice fed LCFAs experienced exacerbated T cell-mediated autoimmune pathological processes (71). Taken together, SCFAs may regulate host immune homeostasis by mediating the differentiation of CD4^+^ T cells to Treg cells.

Primary bile acids (BAs) are synthesized in hepatocytes and converted to various gut BAs by gut commensal bacteria (72). Gut BAs are essential as important molecular mediators to maintain a functional colonic RORγ^+^ Treg cell pool via BA receptors (73). In individual’s gut microenvironment, genetic abolition of BA metabolic pathways remarkably reduces colonic RORγ^+^ Treg cell population, whereas recovery of the gut BA pool elevates the level of these Treg cells (73). The underlying mechanism is that BAs have an important effect on T cell differentiation. In effector T cells, isoalloLCA, a derivative of lithocholic acid (LCA), promotes Treg cell differentiation by enhancing OXPHOS and mitochondrial reactive oxygen species, which upregulates FoxP3 through histone acetylation. In contrast, 3-oxoLCA inhibits Th17 differentiation by interacting directly with the transcription factor RORγt (74) (**Figure 3C**). These studies reveal that BAs and their metabolites modulate the balance of Th17 and Treg cells and elucidate part of the mechanism by which gut microbiota mediates the host immune response.

Gut microbiota can produce Trp metabolites via direct Trp transformation as well as the kynurenine (Kyn) pathway. Direct transformation of Trp generates indole derivatives such as indole ethanol (IE), indolelactic acid (ILA) and indoleacetic acid (IAA), while the Kyn pathway produces Kyn and 3-hydroxyanthranilic acid (3-HAA) (75). These metabolites can activate transcription factors, especially aryl hydrocarbon receptor (AhR) to control the differentiation and function of Treg cells. Kyn produced from the bacterial Kyn pathway induces naïve CD4^+^ T cells to differentiate into Treg cells in an AhR-dependent manner (76). Indole derivatives have been shown to regulate Treg cells via the AhR-ligand-Treg axis, skewing the Th17/Treg balance towards Treg cells (77) (**Figure 3B**). Therefore, Trp metabolites produced by gut microbiota can act as transcription factor ligands to regulate Treg cells, which may be crucial in maintaining intestinal homeostasis.

### 4.2 Microbial metabolism affecting immune tolerance may be involved in pre-RA pathogenesis

As previously mentioned, microbial metabolism can regulate host immune homeostasis through regulating Treg cells. Gut microbiota dysbiosis may impact Treg cell-induced immune tolerance by modulating metabolism, thus promoting the progression of pre-RA. The underlying mechanisms are likely associated with the following metabolic pathways.

**Figure 4:**
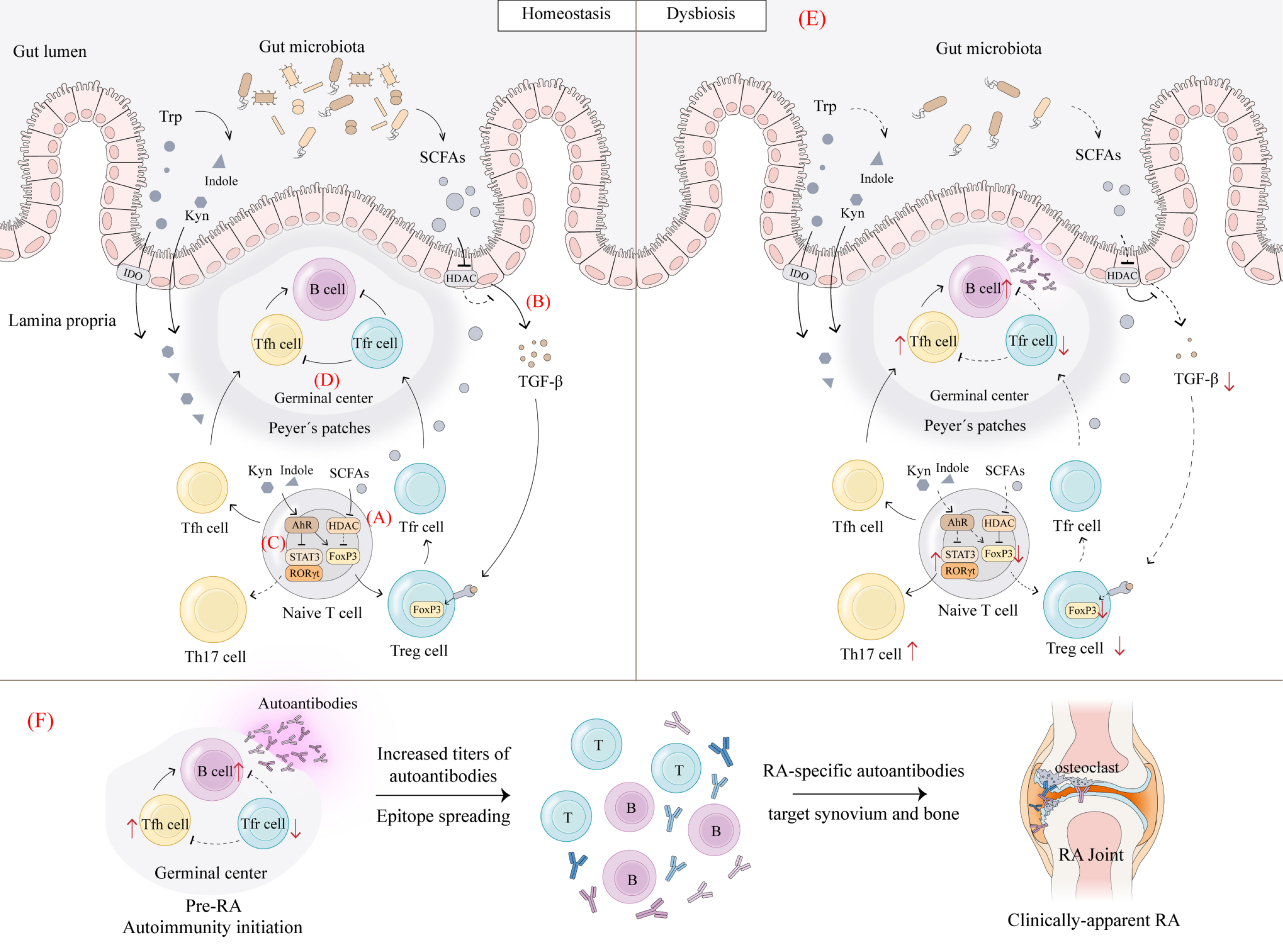
Dysbiosis affects Treg/Th17 and Tfr/Tfh balance. **(A)**, Under homeostasis, SCFAs produced by gut microbiota act as HDAC inhibitors to induce histone H3 acetylation on FoxP3 intronic enhancer, which leads to the expression of FoxP3 in naïve T cells and induces their differentiation to Treg cells (79). **(B)**, Inhibition of HDAC modulates the transforming growth factor β (TGF-β) promoter in intestinal epithelial cells (IECs) and induces the production of TGF-β to promote the *de novo* generation of Treg cells through Smad2/3-induced FoxP3 expression (80). Gut microbiota catabolizes Trp to produce mainly indole and Kyn to activate AhR. **(C)**, The activation of AhR upregulates FoxP3 to increase Treg cell levels and downregulates ROR γ t and STAT3 to decrease Th17 cell levels in order to maintain Th17/Treg balance (84). **(D)**, The activation of AhR also increases the Tfr/Tfh ratio in germinal centers, leading to a decrease in the number of B cells, thus exerting an immunosuppressive effect (85). **(E)**, Upon dysbiosis, SCFAs and Trp metabolites are reduced, thereby decreasing the above signaling-mediated Treg cell and Tfr cell production, leading to Th17/Treg and Tfh/Tfr imbalance and immune tolerance disruption. **(F)**, Initiation of pre-RA autoimmunity followed by increased antibody titers and epitope spreading. As innate and adaptive immune responses amplify, RA-specific autoantibodies target synovial and bone compartments, triggering local inflammatory responses and ultimately developing clinically-apparent RA. *IDO, indoleamine 2,3-dioxygenase. (Created with Adobe Illustrator)*.

SCFAs produced by microbial metabolism have been proven in animal models as key modulators of the gut-joint axis. In collagen-induced arthritis (CIA) mice, butyric acid treatment increased the number of Treg cells and decreased the number of Th17 cells, thereby promoting the restoration of immune homeostasis and effectively inhibiting the progression of arthritis (78). The specific mechanism may involve that SCFAs, including butyric acid can act as HDAC inhibitors to induce histone H3 acetylation on FoxP3 intronic enhancer, which leads to the expression of FoxP3 in naïve T cells and induces their differentiation to Treg cells (79). In addition, the inhibition of HDAC also modulates the transforming growth factor β (TGF-β) promoter in intestinal epithelial cells (IECs) and induces the production of TGF-β to promote the *de novo* generation of Treg cells through Smad2/3-induced FoxP3 expression (80) (**Figures 4A, B**). During the period of CIA induction, Tajik et al. revealed that alterations in the composition of the microbiota, accompanied by a decrease in butyrate levels and impaired intestinal barrier function, occur before the onset of arthritis in mice (81). This provides evidence for the involvement of microbial metabolism in pre-RA pathogenesis. Moreover, the results from Martinsson et al. on individuals at increased risk of RA suggest that SCFAs, particularly butyrate and acetate, can influence the risk of transitioning from the autoimmune to the clinical stage of RA, further demonstrating the role of SCFAs in the pre-RA stage (82).

Trp metabolism may be a key pathway by which gut flora disorders affect Treg cell-induced immune tolerance. A study has identified similar features of disordered Trp metabolism in CIA mice and RA patients, including reduced levels of indole derivatives and Kyns (83). Further administration of recombinant AADAT to modulate endogenous Trp metabolism effectively ameliorated arthritis in mice (83). Trp metabolites may attenuate RA inflammation primarily by activating the transcription factor AhR as its ligand, which in turn affects the induction of CD4^+^ T cells. On the one hand, the activation of AhR can upregulate FoxP3 to increase Treg cell levels and downregulate ROR γ t and STAT3 to decrease Th17 cell levels in order to maintain Th17/Treg balance (84) (**Figure 4C**). On the other hand, it was shown that the activation of AhR increased the T follicular regulatory (Tfr) cell/T follicular helper (Tfh) cell ratio in germinal centers (GCs), leading to a dramatic decrease in the percentage and number of B cells, thus exerting an immunosuppressive effect (85) (**Figure 4D**). Therefore, AhR may serve as an important bridge linking Trp metabolism and immune tolerance. However, views on the role of AhR in RA pathogenesis are controversial, with some studies supporting the potential of AhR activation to alleviate arthritis, while others suggesting that elevated AhR expression in RA patients is associated with greater disease severity (86). This may be attributed to the fact that AhR can be activated by different ligands as well as being involved in RA development through different mechanisms. Undoubtedly, Trp metabolism and its activation of downstream AhR signaling play unique roles in regulating Treg cell and Tfr cell activity and maintaining immune tolerance. At present, gut flora disorders and abnormal Trp metabolism have been observed in patients with new-onset RA and are associated with immune tolerance disruption mediated by decreases in Treg and Tfr cells (87). This suggests the involvement of microbial metabolism in RA pathogenesis by affecting Treg and Tfr cells. Moreover, abnormal Trp metabolism has also been observed in pre-RA patients, indicating its involvement in triggering early mucosal immune imbalance (88).In summary, gut flora disorders may lead to a decrease in relevant Trp metabolites, which in turn reduces the production of Treg and Tfr cells mediated by AhR signaling, leading to a disruption of immune tolerance, followed by increased antibody titers, epitope spreading, and RA-specific autoantibody production, and ultimately the development of clinically-apparent RA (**Figures 4E, F**).

As previously stated, the main alterations in the composition of gut microbiota in RA have been an expansion of *Prevotella copri* and a decline in *Bacteroides*. Several studies have suggested potential pathways by which they affect host metabolism. Su et al. demonstrated that excessive *Prevotella copri* can consume a significant amount of Trp without the production of indole-3-pyruvic acid (IPyA) (an activator of AhR) (89). It can therefore be hypothesized that increased *Prevotella copri* in RA may also consume Trp without producing AhR activators through specific pathways, thus preventing the physiological accumulation of activators such as Kyn and the subsequent activation of AhR, which in turn affects FoxP3 expression, leading to reduced population and impaired function of Treg cells. *Bacteroides* have been found to reduce inflammation through the production of propionic acid, which may promote Treg cell development through HDAC-dependent induction of FoxP3 (90). However, there is a lack of conclusive evidence for the association between RA-related gut microbiota and metabolic disorders. Further investigation is required to identify the characteristic gut microbiota that manipulates key metabolisms in RA and the specific mechanisms affecting Treg cells.

## 5 Treg cells participate in shaping and protecting the gut microenvironment

**Figure 5:**
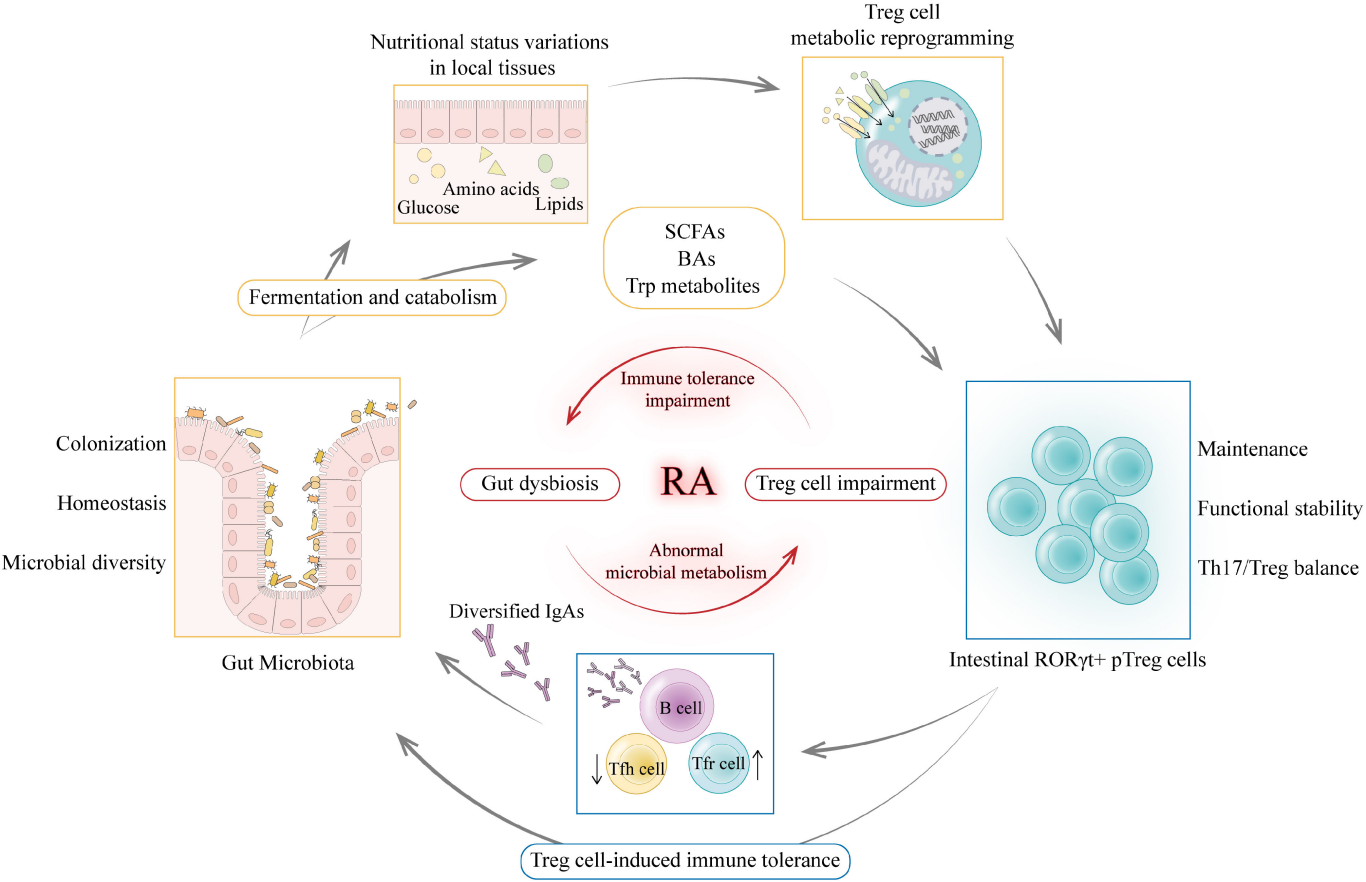
Immunometabolic dialogue between gut microbiota and Treg cells. The interaction between gut microbiota and Treg cells is a two-way street. On the one hand, gut microbiota catabolism of macronutrients and complex dietary fibers can alter nutritional status in local tissues to drive metabolic reprogramming of Treg cells thereby maintaining a functional intestinal RORγ^+^ pTreg cell pool. Gut microbiota metabolites such as SCFAs, BAs and Trp metabolites can regulate intestinal RORγ^+^ pTreg cell levels and affect Th17/Treg balance. On the other hand, as key metabolic sensors, Treg cells can translate the above metabolic signals into immune tolerance regulation. Treg cells maintain tolerance to commensal flora and regulate the selection of immunoglobulin A (IgA) in germinal centers. Diversified and selected IgAs support a diverse and balanced microbiota in the gut (92). Thus, Treg cells guarantee stable colonization and diversity of the gut microbiota through immune signals. When individuals suffer from gut dysbiosis, abnormal microbial metabolism affects Treg cell expression and function, which in turn impairs the regulation and maintenance of gut microbiota, exacerbating gut immune disorders, potentially creating a vicious cycle that amplifies inflammation and drives the progression of RA. *(Created with Adobe Illustrator)*.

Gut Treg cells are essential for the maintenance of homeostasis in the gut microenvironment and are particularly involved in shaping and protecting the ecology of the gut microbiota (20). The interaction between gut microbiota and Treg cells is a two-way street. Gut microbiota metabolism regulates Treg cell homeostasis, while Treg cells also exert an important regulatory role on gut microbiota composition. In mice lacking FoxP3 CNS1, which is essential for pTreg cell induction in gut microenvironment, the relative abundance of the phylum Firmicutes in the gut is reduced (91). Treg cells contribute to the diversity of the commensal microbiota as well. Immunoglobulin A (IgA) production and selection in the GC of Peyer’s patches is supported by Treg cells. The diversified IgA supports a diverse microbiota in the gut, especially the diversity of Clostridia cluster IV and XIVa (92). More importantly, such regulation of microbial diversity by Treg cells is necessary for mucosal immune system maturation. Therefore, a regulatory circuit is formed between Treg cells and gut microbiota through immunometabolic dialogue to maintain host-microbe symbiosis and immune homeostasis. Using the DEpletion of REGulatory T cells (DEREG) mouse model, Kehrmann et al. found alterations in the composition of the gut microbiota before and after Treg cell depletion, accompanied by an increase in gut inflammation, suggesting a role for Treg cells influencing gut microbiota in the maintenance of intestinal homeostasis (93). Although the impact of Treg cell changes on gut microbiota has not been investigated in RA-related models, it can be hypothesized that when individuals suffer from gut dysbiosis, abnormal microbial metabolism affects Treg cell expression and function, which in turn impairs the regulation and maintenance of gut microbiota, exacerbating gut immune disorders, potentially creating a vicious cycle that amplifies inflammation and drives the progression of RA (**Figure 5**).

It is clear that we are just beginning to comprehend the close relationship between Treg cells and gut microbiota, and much remains to be discovered about the interactions between microbial metabolism and the host immune system. Thus, further investigation into the characteristics of distinct gut Treg cell subpopulations and the immunometabolic dialogue between gut microbiota and Treg cells will undoubtedly facilitate the development of more targeted intervention strategies. These strategies may potentially offer new therapeutic opportunities based on Treg cells, gut microbiota, and metabolites for RA.

## 6 Restoring immune tolerance based on Treg cells, gut microbiota, and metabolic pathways

Currently, conventional synthetic, targeted, and biologic disease-modifying anti-rheumatic drugs (DMARDs) are widely used to control RA progression, but almost all of them are immunologically non-selective without tackling the fundamental immune mechanisms of RA (94). RA patients tend to take various DMARDs long-term, increasing the risk of infections, tumors, and other adverse effects. Therefore, it is reasonable to fundamentally restore immune homeostasis in RA patients by remodeling immune tolerance. Moreover, the prevention of RA has been a major clinical concern and the pre-RA stage may be an ideal window for intervention. Early detection of RA-related autoimmunity and reestablishing immune tolerance in pre-RA individuals may achieve desirable results. Considering that the immunometabolic dialogue between gut microbiota and Treg cells may play a key role during the pre-RA phase, targeting Treg cells, gut microbiota, and metabolic pathways could offer new therapeutic options.

### 6.1 Treg cell therapy

Due to their critical role as regulators of immune homeostasis, Treg cells are ideal candidates for restoring immune tolerance in RA. Current therapies based on Treg cells include *in vitro* expansion of Treg cells for adoptive transfer and administration of immunomodulatory interventions to promote *in vivo* Treg cell expansion and/or function (95). In particular, low-dose IL-2 therapy may have the potential to modulate immunometabolic pathways.

Treg cell differentiation, function and maintenance rely on IL-2. The dose of IL-2 affects the balance between immune tolerance and autoimmunity. High doses of IL-2 promote autoimmunity by activating effector T cells, whereas low doses of IL-2 induce only the development of Treg cells but not effector T cells (96, 97). Considering its different roles in modulating Treg and Th17 cell differentiation, low-dose IL-2 could provide an important therapeutic approach for targeting Treg and Th17 cells in RA. Compared to biologics targeting pro-inflammatory factors such as anti-TNF, low-dose IL-2 therapy mitigates autoimmune disease activity at a higher level by modulating the Th17/Treg balance, effectively decreasing pro-inflammatory factors (98). Current studies have provided clinical evidence for the safety, biological and clinical effects of low-dose IL-2 in RA (99, 100). Moreover, in CIA mice, low-dose IL-2 treatment improved gut microbiota dysbiosis and SCFA-related metabolic disturbances, significantly alleviating arthritis, suggesting its potential in modulating immunometabolism (101). Future exploration of the broader therapeutic effects and long-term efficacy of low-dose IL-2 may provide new prospects for restoring pre-RA immune homeostasis.

### 6.2 Therapies targeting gut microbiota

A variety of beneficial bacteria have been found to have immune regulatory effects in animal models, thereby reducing arthritis. For example, *L. casei* has been proven to reduce the development and progression of adjuvant-induced arthritis (AIA) by reestablishing gut microbiota homeostasis (102). The osteoprotective effect of *Lactobacillus rhamnosus* by regulating Th17/Treg balance has been confirmed in research on bone-related diseases (103). A therapeutic bacterial strain, *P. histicola* MCI 001, has been demonstrated to protect CIA mice from arthritis by increasing the production of SCFAs (104). However, the evidence for the practical effectiveness of probiotics in improving arthritis in RA patients remains limited. Hatakka et al. evaluated the effects of *Lactobacillus rhamnosus* supplementation in RA patients without receiving DMARDs. Compared to the placebo group, no significant differences in inflammatory markers or clinical disease status were found, but patients receiving probiotic supplementation reported subjective improvements (105). Although no remarkable therapeutic effects have been found with probiotics in established RA, based on the role of the microbiome in RA pathogenesis and its alterations in RA patients, probiotics may serve as an adjunctive therapy for RA or could be effective in pre-RA. Additional research is required to evaluate the potential and safety of probiotic treatments in the clinical management of RA.

Besides, gut microbiota and associated metabolism can be intervened with various measures such as diet, plant extracts, prebiotics, and fecal microbiota transplantation (106). Extensive meta-analyses have suggested that oral supplementation with omega-3 polyunsaturated fatty acids dramatically reduces levels of inflammation-associated markers through the modulation of gut microbiota (107). The natural compound ginsenoside Rg1 could increase serum levels of Trp metabolites, mainly indole derivatives (108). It markedly changed the gut microbiota composition and serum Trp in high-fat diet-induced obese mice (109). Some chemicals can also modulate Trp metabolism mediated by gut microbiota, such as Fisetin (110). Considering that abnormal Trp metabolism may be one of the important mechanisms triggering autoimmunity in pre-RA, relevant metabolic drugs may have the potential for early intervention in high-risk individuals. Dietary interventions such as a high-fiber diet in RA patients could result in increased levels of circulating Treg cells, favorable Th1/Th17 ratios, and a reduction in bone erosion markers. This may be because ingested dietary fibers can be fermented by gut microbiota to produce SCFAs, which exert immune regulatory functions (111). Additionally, consuming a high-fiber bar daily might be easy for RA patients to maintain and be well accepted by pre-RA patients. Therefore, high-fiber dietary interventions may serve as a viable approach to complement existing pharmacological therapy strategies for RA. Along with high-fiber diets, improvements in RA have also been seen in studies of other dietary interventions, such as the Mediterranean diet and supplementation with vitamin D and omega-3 fatty acids (112, 113). So far, however, none of these dietary interventions have been widely implemented in clinical practice. This may reflect a lack of understanding of the underlying immunometabolic mechanisms of dietary interventions in RA, or that the effectiveness of existing DMARDs overshadows the adjunctive role of nutritional supplements. The therapeutic and preventive potential of dietary interventions in RA deserves further evaluation.

### 6.3 Therapies targeting metabolic pathways

Restoring immune homeostasis by regulating metabolic procedures is promising and may open up new choices for repurposing existing metabolic drugs. Rapamycin may restore immune tolerance by influencing the metabolic reprogramming of Treg cells (114). mTORC1 suppression by rapamycin induces FAO, inhibits Teff cell proliferation, and promotes Treg cell generation (115). Moreover, research suggests that rapamycin therapy in mice with autoimmune symptoms reverses their inflammatory phenotype, recovers Treg cell function, and extends their lifespan (116). Metformin, the most commonly used antidiabetic drug, can broadly affect metabolic pathways and immune functions (117). Specifically, metformin can induce FAO through AMP-activated protein kinase (AMPK) activation, thereby regulating Treg cell production (39). Studies have demonstrated that the administration of metformin in murine models of autoimmune diseases induces the generation of Treg cells while inhibiting the differentiation of Th17 cells, which reduces disease burden in mice (118, 119). In addition, studies have shown that metformin can alter the composition of the gut microbiota and increase circulating SCFAs, which are associated with its therapeutic effects on type 2 diabetes (120, 121). Therefore, metformin may also influence Treg cells by regulating microbial metabolism, thereby contributing to the restoration of immune homeostasis. Acarbose has been reported to alleviate CIA by modulating the Th17/Treg axis in gut mucosal immunity, possibly due to its effect on gut microbiota (122). To date, several reports have demonstrated the safety and efficacy of metformin and acarbose as adjuvant therapies for RA, but most were based on animal studies and retrospective clinical trials (122, 123). Therefore, further studies are needed to elucidate their practical efficacy in RA. Moreover, few studies have focused on the relationship between metabolic drugs and the risk of RA, thus, their effectiveness in the pre-RA stage and RA prevention requires further investigation.

Interventions based on immune metabolism and gut microbiota to regulate immune homeostasis may guide a new direction for RA treatment and prevention. Future deciphering of the immunometabolic dialogue between gut microbiota and Treg cells to explore the specific mechanisms of the transition from pre-RA to clinically-apparent RA will facilitate the identification of novel therapeutic targets for early restoration of immune tolerance.

## 7 Discussion

The past decade has seen an unprecedented breadth and depth of exploration of RA pathogenic mechanisms. Advances in treatment have made remission an achievable goal for many patients, nevertheless, RA remains a lifelong incurable disease. Patients usually require long-term or even lifelong use of various DMARDs, which increases the risk of adverse effects such as infections and tumors. Therefore, the next challenge in RA management is to maintain remission with a minimal therapeutic regimen, or even to achieve the ideal state of drug-free remission. Restoring immune tolerance could be an attractive strategy because of its potential to intervene in the underlying immune mechanisms that initiate RA. It is now generally accepted that the initial disruption of immune tolerance occurs at a stage long before clinically-apparent IA, known as the pre-RA phase, and may begin with the failure of Treg cell-induced immune tolerance to gut microbiota. More in-depth studies of the complex immunometabolic interactions between Treg cells and gut microbiota will help to unravel the biological pathways specific to pre-RA and identify key markers for the early identification of at-risk individuals. Moreover, the introduction of the pre-RA concept shifts the aim of RA prevention from preventing more severe joint injuries in established RA patients to preventing the first onset of clinically-apparent IA. Accurate prediction of future RA is a key aspect of prevention, but there exist many challenges. Currently, most prospective studies recruit subjects with joint symptoms and have difficulty recruiting individuals in the pre-RA state as they do not seek treatment. If we expect asymptomatic individuals to participate, additional efforts will be needed to educate the public extensively about what screening and prevention of RA could mean for their health. In the future, more appropriate intervention targets for the pre-RA phase may be identified from an immunometabolic perspective. Treatments based on immunometabolic mechanisms such as the administration of probiotics, prebiotics, and nutritional supplements may be more acceptable to high-risk asymptomatic individuals.
